# Fatigue Damage Behaviors of TRC-Strengthened RC Beams

**DOI:** 10.3390/ma15155113

**Published:** 2022-07-22

**Authors:** Jie Sheng, Zongjian Yu, Guotao Dou, Hao Liu

**Affiliations:** 1 School of Civil Engineering, Xuzhou University of Technology, Xuzhou 221000, China; 2 Jiangsu Key Laboratory of Environmental Impact and Structural Safety in Engineering, School of Mechanics and Civil Engineering, China University of Mining and Technology, Xuzhou 221116, China; zongjian_yu@cumt.edu.cn; 3 School of Civil Engineering, Zhengzhou University of Aeronautics, Zhengzhou 450046, China; zzhydgt@zua.edu.cn; 4 Xuzhou Construction Engineering Testing Center Co., Ltd., Xuzhou 221000, China; zhu881231@gmail.com

**Keywords:** bending fatigue, damage behavior, textile reinforced concrete, strengthened, RC beam

## Abstract

For the investigation of fatigue damage behavior of textile reinforced concrete (TRC)-strengthened RC beams, in this study, eight RC beams were fabricated, and five of them were strengthened with TRC and tested under fatigue loading until failure, using a four-point bending setup. Research parameters included reinforcement ratio, textile ratio, and strengthening methods (single-side and U-wrapped). The failure mode, fatigue life, fatigue deformation, and other properties of TRC-strengthened beams were analyzed. Experimental results revealed that there were two fatigue failure modes for TRC-strengthened RC beams. In the first mode, the textile was snapped, but the steel bars did not rupture. In the second mode, both the textile and steel bars broke. Fatigue failure modes depended on the textile ratio. The TRC-strengthened beam’s fatigue life was significantly higher than the non-strengthened RC beam. At the same textile ratio, the TRC-strengthened beam’s fatigue life using the single-side method was longer than that using the U-wrapped method. With the increase in fatigue loading time, the midspan deflection of the TRC-strengthened beam was developed in three stages, namely rapid development stage, stable development stage, and destabilized development stage. The residual deflection and strain damage accumulation of tensile steel bars of TRC-strengthened beams were significantly reduced with the increase in textile or reinforcement ratios; thus, the beam’s fatigue life was prolonged.

## 1. Introduction

Reinforced concrete bridges are extensively employed due to the simplicity of their construction and low cost. In China, reinforced concrete (RC) bridges comprise more than 90% of the total number of existing bridges. However, as their service time is increased, RC bridges become susceptible to fatigue damage under long-term alternating loads, and with the accumulation of fatigue damage, the collapse risk of RC bridges is increased. Strengthening can be considered an efficient approach for improving RC bridge fatigue performance. Nie et al. [1] conducted fatigue experiments on eight RC beams strengthened with the steel plate–concrete composite (SPCC) technique. The results showed that the fatigue stiffness of RC beams can be improved by using the SPCC technique, and the effective improvement of fatigue life can be achieved. However, when steel plates are used to strengthen RC bridges, the corrosion problem is hard to avoid, especially in some harsh environments. Fiber-reinforced polymers (FRPs) can solve the corrosion problem of steel plates and have been extensively applied for strengthening concrete structures owing to their corrosion resistance, light weight, high strength, and ease of construction [2,3,4]. Zhu et al. [5] found that when carbon-fiber-reinforced plastics (CFRPs) are applied to strengthen RC beams, the fatigue damage accumulation rate of the RC beams can be effectively reduced. If a prestressed CFRP was adopted to strengthen the RC beam, a better effect could be reached in terms of fatigue resistance. Although the FRP strengthening technique has many advantages, FRP composites mainly apply epoxy resin as binder, which suffers from the disadvantage of easy aging of binder material, easy debonding from the surface of the original structure, and difficulty of construction on wet surfaces [6,7,8].

Currently, a new strengthening material, i.e., textile-reinforced concrete (TRC), has attracted the attention of researchers. This is a composite material in which high-performance fine concrete is used as the matrix, and textile acts as the bearing material [9,10,11,12]. When using TRC for strengthening concrete structures in situ, high-performance fine concrete is employed as the binder to bond textile onto the concrete component’s surface. Therefore, the disadvantages of the FRP strengthening method caused by the binder could be overcome. The textile-reinforced mortar (TRM) and fabric-reinforced cementitious matrix (FRCM) are composite materials of the same type as TRC and are considered different terms for referring to TRC.

The effectiveness of TRC in improving the bearing capacity of concrete structures has been confirmed in several research works [13,14,15,16,17,18,19]. However, research on the fatigue performance of TRC-strengthened RC beams is insufficient. Pino et al. [20] and Hadad et al. [21] used polyparaphenylene benzobisoxazole (PBO) and carbon fabric-reinforced cementitious matrices (PBO–FRCM and C-FRCM, respectively) for strengthening RC beams and the influence of the number of fabric layers and stress level on the fatigue performance of strengthened beams were evaluated. Research findings revealed that FRCM can effectively control fatigue crack development, improve fatigue crack distribution, and significantly increase fatigue life. Aljazaeri et al. [22] studied the fatigue performance of PBO–FRCM-strengthened RC beams after freeze–thaw cycles. Their results showed that the fatigue life of the FRCM-strengthened beam was reduced due to the decrease in mechanical properties and bonding performance between steel and concrete caused by the freeze–thaw cycle [23]. However, the decreasing rate of the fatigue life of FRCM-strengthened beams was slower than that of the non-strengthened beams. Elghazy et al. [24] and Bressan et al. [25] studied the fatigue performance of corrosion-damaged RC beams strengthened by various FRCM types. Their results revealed that the fatigue lives of rusted RC beams were increased by 38–377%, depending on fabric amount and FRCM type. Based on existing studies, FRCM is considered to effectively enhance RC beam fatigue performance. On the other hand, the fatigue damage behaviors of FRCM-strengthened RC beams are still poorly understood. Additionally, existing studies have only used bare textiles, resulting in the premature failure of fiber filaments in textiles due to repeated friction between the textile and the matrix caused by cyclic loading [26]. This phenomenon may limit the fatigue performance of strengthened beams.

Based on the above analyses, to further study the fatigue behaviors of TRC-strengthened RC beams, this study used impregnated and sand-bonded textiles to improve synergistic bearing performance among fiber filaments and enhance the bonding capacity between the matrix and the textile. Then, a four-point bending fatigue test was conducted to investigate the influences of the strengthening method, textile ratio, and reinforcement ratio on the fatigue damage behavior of TRC-strengthened beams.

## 2. Materials and Methods

### 2.1. RC Beam Design

**Figure 1:**
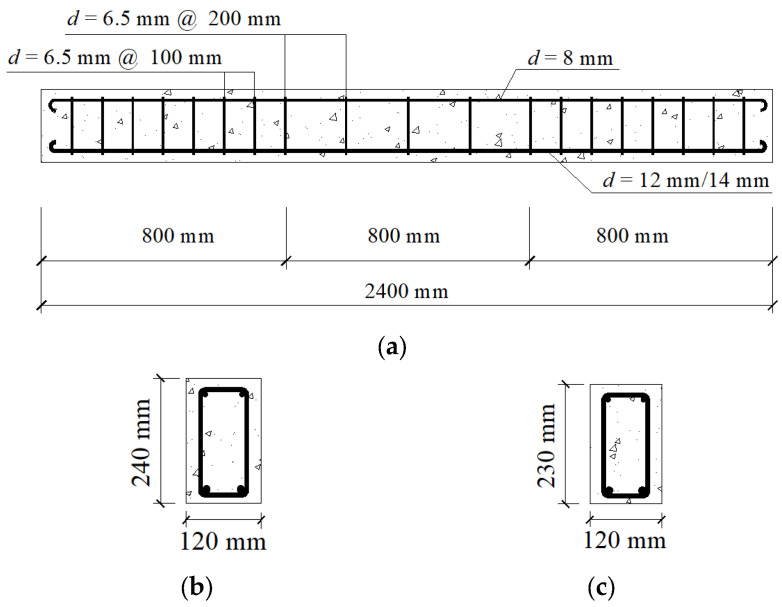
Reinforcement and geometry of RC beams: (**a**) steel bar arrangement; (**b**) cross-section of RC non-strengthened beams; (**c**) cross-section of RC beams strengthened with TRC.

Six RC beams were used for fatigue tests. One of these beams was not strengthened and acted as a reference beam, and five beams were strengthened with TRC. Besides these beams for fatigue tests, two RC beams were fabricated for static tests to determine fatigue load. The dimensions of the reference beam applied in the fatigue test and those used in static tests were 120 × 240 × 2400 mm, and the dimensions of RC beams that would be strengthened with TRC were 120 × 230 × 2400 mm. The diameter of the tensile ribbed steel bar was 12 or 14 mm. Plain round bars 8 and 6.5 mm in diameters were used as erection bars and stirrups, respectively. Stirrup spacing in pure bending and shear span sections were 200 and 100 mm, respectively. The reinforcement and geometry of RC beams are shown in Figure 1.

### 2.2. Strengthening Scheme

**Figure 2:**
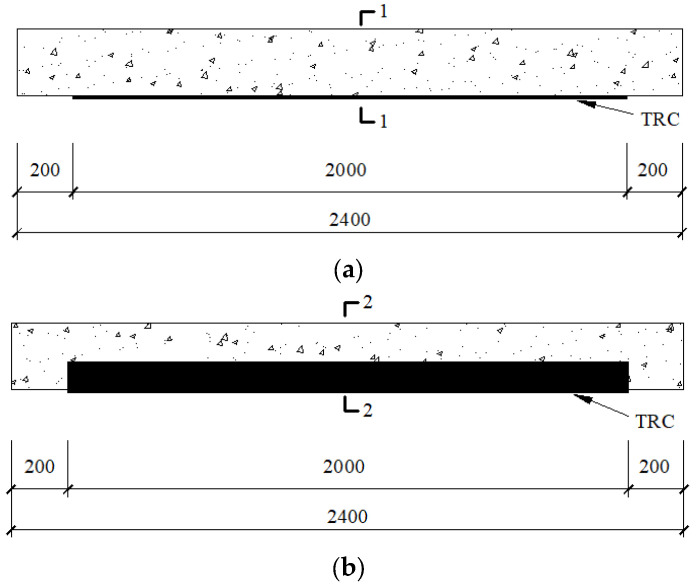

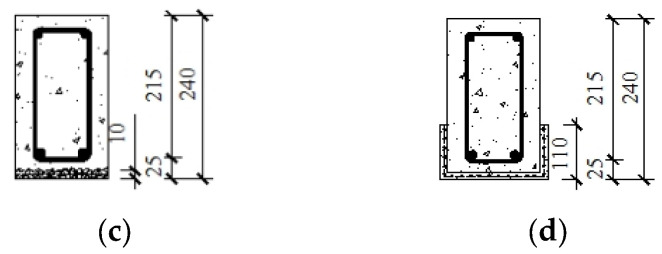
Strengthening schemes diagram (unit: mm): (**a**) side view of the beam using single-side method; (**b**) side view of the beam using U-wrapped method; (**c**) 1-1; (**d**) 2-2.

Two different strengthening methods were adopted in this work: the single-side method and the U-wrapped method. The single-side technique meant that the TRC was only arranged at the RC beam bottom, while in the U-wrapped method, the TRC was placed both at the bottom and on the sides of the RC beam. The TRC layer’s thickness was 10 mm. The textile arranged at the RC beam bottom was 2000 mm in length and 120 mm in width (containing 10 carbon fiber bundles). The textile placed on the beam side was 2000 mm in length and 60 mm in width. Figure 2 shows the specific schematic diagram of strengthened RC beams.

An “M/F-0/BP/UP-D” naming format was used for the specimens, where M denotes the static test, F denotes the fatigue test, 0 denotes non-strengthened by the TRC, B denotes the single-side method, U denotes the U-wrapped method, P is the textile layer number, and D is the tensile rebar’s diameter. For example, F-U2-14 referred to the specimen strengthened by the U-wrapped method, with two textile layers and a diameter of 14 mm. The specific information of the specimens is presented in Table 1.

### 2.3. Materials

#### 2.3.1. Concrete

In this work, the concrete ratio of 1:0.47:1.55:2.85:0.022 for cement:water:sand:gravel:water reducing admixture was adopted. P.O. 42.5 Portland cement (a type of commercial cement in China) was used along with ordinary river sand with a fineness modulus of 2.7, the maximum gravel particle size of 10 mm, and a polycarboxylate superplasticizer water-reducing admixture. Based on the *Standard for the test methods of concrete’s physical and mechanical properties (GB/T 50081-2019)*, three cubic specimens with 150 × 150 × 150 mm dimensions were evaluated to determine compressive strength after 28 days of standard curing. The average measured concrete compressive strength was 44.6 MPa.

#### 2.3.2. Steel Bar

To obtain the uniaxial tensile mechanical behaviors of the tensile steel bar applied in the current research, tensile specimens were loaded using a UTM5305 g electronic universal testing machine. The tensile properties of tensile steel bars were tested based on *Metallic Materials—Tensile Testing-Part 1: Method of Test at Room Temperature (GB/T228.1-2010)*. Table 2 summarizes the mechanical properties of steel bars.

#### 2.3.3. Textile

**Figure 3:**
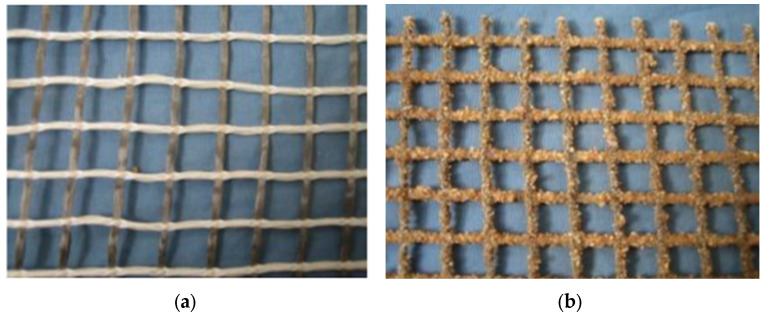
Original and sand-bonding textile: (**a**) original textile; (**b**) after sand-bonding.

In the current research, a hybrid textile comprising E-glass and carbon fibers (carbon fiber and glass fiber were made by Toray corporation, Tokyo, Japan) was applied, as presented in Figure 3a. The textile mesh size was 10 × 10 mm. A carbon fiber bundle with a cross-sectional area of 0.45 mm^2^ was used to bear tensile stress. Glass fiber bundle was used for fixing the carbon fiber bundle. Before application, epoxy resin (the type was E-44, the manufacture is Nantong Xingchen Synthetic Material Co., Ltd., Nantong, China) was used to impregnate the textile to enhance the cooperative bearing ability among fiber filaments in bundles. Subsequently, sand-bonding treatment was adopted for improving the bonding properties between the matrix and the textile. Figure 3b shows the textile obtained after sand-bonding. The mechanical properties of the fiber yarns of textile are shown in Table 3.

#### 2.3.4. Matrix of TRC

The TRC matrix was a cementitious material called high-performance fine concrete and consisted of P.O. 52.5R ordinary Portland cement, coarse sand (26–32 mesh common quartz sand), fine sand (32–64 mesh common quartz sand), first-class fly ash (FAI), and a polycarboxylate superplasticizer. High-performance fine concrete has high fluidity, and its actual compressive strength, as measured by tests on cubes with 70.7 × 70.7 × 70.7 mm dimensions, was 52.8 MPa at 28 days. Table 4 lists the proportions of the high-performance fine concrete mix.

### 2.4. Loading Scheme

**Figure 4:**
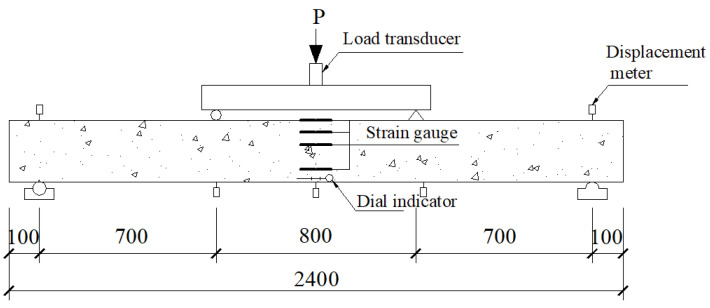
Loading and measuring point layout schematic diagram (unit: mm).

Figure 4 shows the measuring and loading point configurations. A fatigue load of equal amplitude sinusoidal function with a frequency of 3 Hz was applied. The maximum (*F*_max_) and minimum (*F*_min_) fatigue loads were 0.7 *P*_u_ and 0.2 *P*_u_, respectively, where *P*_u_ is the static ultimate load of tested beams before strengthening. Specimens M-0-12 and M-0-14 were subjected to static loading to obtain the ultimate load (*P*_u_). The yield load (*P*_y_) of specimens M-0-12 and M-0-14 were 63.10 kN and 80.12 kN, respectively. The ultimate load (*P*_u_) of specimens M-0-12 and M-0-14 were 69.78 kN and 85.00 kN, respectively.

A dynamic hydraulic servo instrument (Popwill corporation, Hangzhou, China) was applied for fatigue tests. The maximum load it can provide is 200 kN, and the rated frequency from ranges 0 Hz to 50 Hz. Before fatigue loading, a static load was applied to the tested beams from zero to the maximum fatigue load using the step-loading method to eliminate the gap in the test system. Then, the tested beams were unloaded to zero, and fatigue tests began. During the fatigue loading progress, when the number of cycles reached 1 × 10^3^, 5 × 10^3^, 1 × 10^4^, 1.5 × 10^4^, 2 × 10^4^, 3 × 10^4^, 4 × 10^4^, 6 × 10^4^, 1 × 10^5^, 1.5 × 10^5^, 2.5 × 10^5^, 3 × 10^5^, 4 × 10^5^, and 5 × 10^5^, the machine was halted. Then, static loading was applied from zero to the maximum fatigue load using the step-loading method. During this process, the residual deflection, midspan deflection, concrete strain, and steel bar strain of the tested beams were automatically recorded using a data acquisition instrument. A crack-width gauge was applied to measure crack width. Tests were completed when a fatigue failure was detected.

## 3. Results and Discussion

### 3.1. Failure Modes

**Figure 5:**
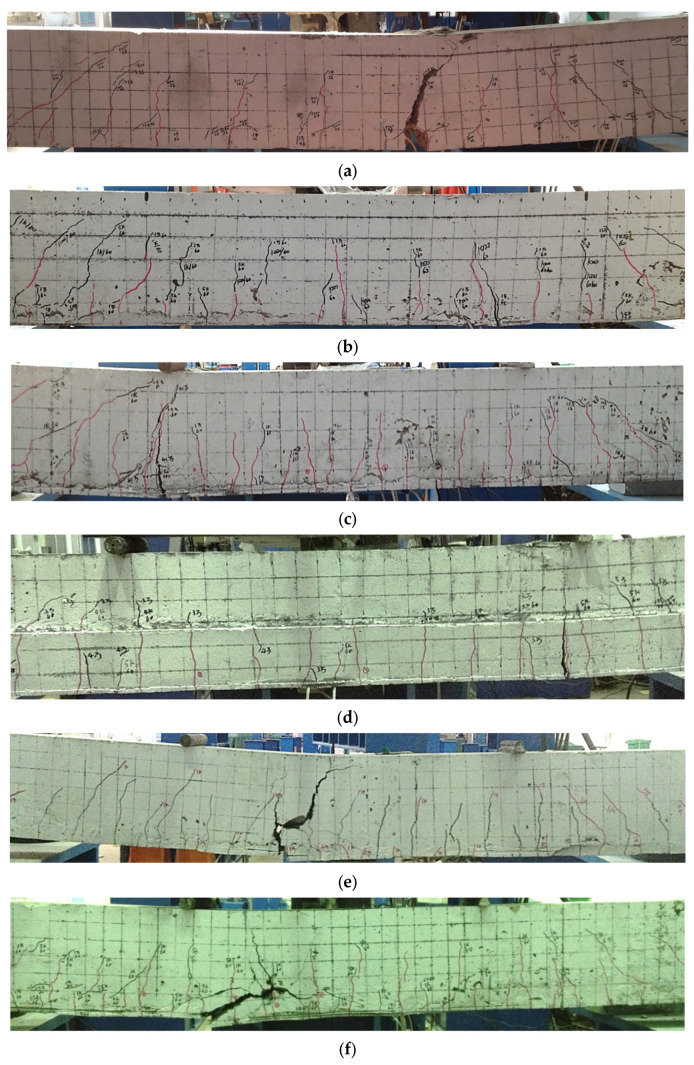
Failure pattern of each specimen: (**a**) F-0-14; (**b**) F-B1-14; (**c**) F-B2-14; (**d**) F-U2-14; (**e**) F-B3-12; (**f**) F-B3-14.

The fatigue cracks of TRC-strengthened beams had similar development trends, which indicated that fatigue cracks exhibited three-stage development during fatigue loading. First, the rapid development stage occurred. In this stage, the TRC-strengthened beam cracks were increased and rapidly propagated to the compression zone as the number of cycles increased. This stage was finished when 30,000–40,000 cycles were reached. The second stage was the stable crack-propagation stage, during which the cracks propagated at a much slower rate than in the first stage. Additionally, in this stage, the number of cracks was almost the same as that in the first stage. The third stage began when crack propagation became unstable. In this stage, the cracks’ width and size were increased, and a distinct primary crack appeared, which indicated that the TRC-strengthened beam was about to be damaged. Figure 5 illustrates the final failure situation of each tested beam. As observed in Figure 5, the crack number in TRC-strengthened beams was significantly higher than that in non-strengthened beams, indicating the critical role of TRC in crack dispersion.

From the test results, two different fatigue failure modes were witnessed for TRC-strengthened beams. In the first mode, concrete in the compression zone was crushed, and the textile snapped, but the tensile steel bar did not rupture. This fatigue failure mode was observed in specimens F-B1-14, F-B2-14, and F-U2-14. The common feature of these three beams was that their textile ratios were less than 0.036%, which led to wide stress variations in carbon fiber bundles during the fatigue loading progress. When the textile snapped, the TRC-strengthened beam’s deflection was suddenly increased and the neutral axis position was high, which caused concrete crushing in the compression zone. As tensile steel bars could bear the stress transmitted by the textile fracture, they did not rupture.

In the second fatigue failure mode, the textile snapped, and one tensile steel bar ruptured, but the concrete in the compression zone was not crushed. The fracture of the steel bar is the main reason for this failure mode. When tensile steel bars reached fatigue life, and a fatigue fracture occurred, the tensile stress was released and transferred to the textile, which led to the textile fracture owing to the relatively low bearing capacity of carbon bundles. This failure mode was observed in F-B3-12 and F-B3-14 specimens. These two beams had a relatively high textile ratio (ρt ≥ 0.036%), which resulted in lower neutral axis positions in TRC-strengthened beams even under fatigue failure. Therefore, in this fatigue failure mode, the compression zone concrete was not crushed.

From the above analyses, it was derived that the TRC-strengthened beam fatigue failure mode was controlled by textile ratio. The first fatigue failure mode occurred when the textile ratio was ≤0.036%. The second fatigue failure mode occurred when the textile ratio was >0.036%. However, the fatigue failure modes of TRC-strengthened beams must be further studied because this study considered a limited number of specimens.

**Figure 6:**
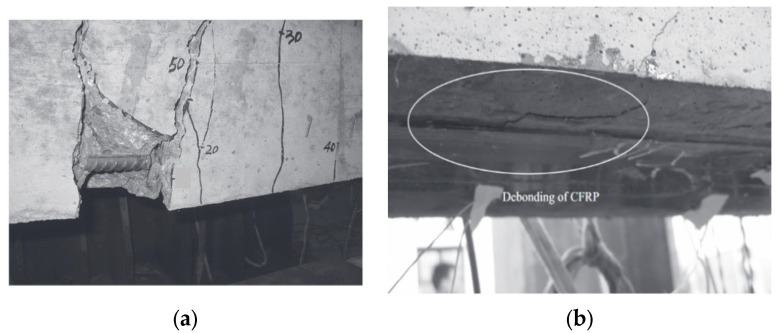
The fatigue failure mode of CFRP-strengthened beam (photos from Ref. [27]): (**a**) fatigue failure; (**b**) CFRP debonding.

Peng et al. [27] studied the flexural fatigue performance of RC beams strengthened with CFRP plates. The test results showed that the CFRP-strengthened beam had larger crack spacing (Figure 6a), compared with the TRC-strengthened beam. Additionally, a debonding phenomenon was observed between the CFRP plate and the concrete during the fatigue test, resulting in stress redistributions between the CFRP plate (Figure 6b), while in the TRC-strengthened-beam, the debonding phenomenon did not occur.

### 3.2. Fatigue Life

Table 5 presents the fatigue lives of tested beams. As can be seen, all TRC-strengthened beams, except F-B1-14, had higher fatigue life than the non-strengthened beam F-0-14. Specimen F-B1-14, which had only one textile layer, had lower fatigue life than F-0-14. The difference between the fatigue lives of these two beams was 19,300 cycles, which is not large. This phenomenon was attributed to the poor fatigue performance of the impregnated textile or to the discrete data. Additionally, the fatigue life of F-B3-14 was 498,000 cycles, which was 1.58 times longer than that of F-0-14. Therefore, it was concluded that TRC could significantly improve the RC beam’s fatigue life when the textile ratio was sufficient.

As presented in Table 5, the fatigue life of specimen F-B2-14 was 415,300 cycles, while that of F-U2-14 was 381,000 cycles. The fatigue life of F-B2-14 was 10% higher than that of F-U2-14. This phenomenon indicated that, when the textile ratio is same, the single-side strengthening method was superior to the U-wrapped strengthening method in terms of fatigue life. For specimen F-U2-14, the stress in lateral carbon fiber bundles was decreased along the direction of beam height; therefore, the stress amplitude of carbon fiber bundles at the bottom of specimen F-U2-14 was greater than that of specimen F-B2-14, which resulted in relatively small fatigue life for specimen F-U2-14.

**Figure 7:**
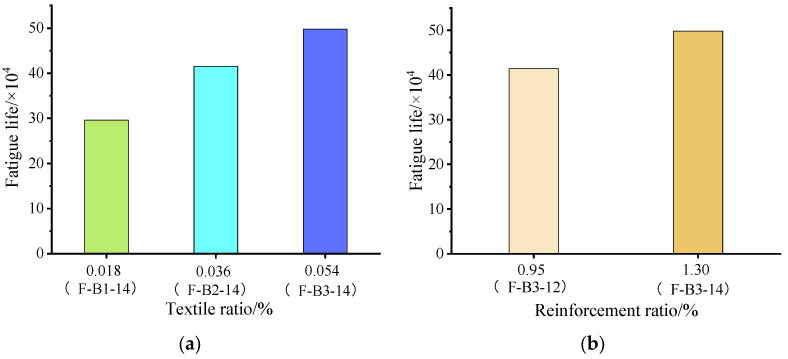
The relationship between textile ratio/reinforcement ratio and fatigue life: (**a**) fatigue life–textile ratio; (**b**) fatigue life–reinforcement ratio.

Figure 7a shows the fatigue life–textile ratio relationship. As is commonly known, the TRC-strengthened beam’s fatigue life increases by increasing the textile ratio. The fatigue life of specimen F-B2-14 was 415,300 cycles, that is, 40.2% higher than that of specimen F-B1-14. The fatigue life of specimen F-B3-14 was 498,000 cycles, which was 20.0% higher than that of specimen F-B2-14. Consequently, although the TRC-strengthened beam’s fatigue life was increased by increasing the textile ratio, the rate of this increase was reduced, possibly due to different failure modes.

Figure 7b illustrates the fatigue life–reinforcement ratio relationship. As was observed, the fatigue life of F-B3-12 was 414,200 cycles, while that of F-B3-14 was 498,000 cycles, which indicated that the fatigue life of F-B3-14 was 20.2% higher than that of F-B3-12, proving that increasing reinforcement ratio improved fatigue life.

### 3.3. Midspan Deflection Corresponding to Maximum Fatigue Load

**Figure 8:**
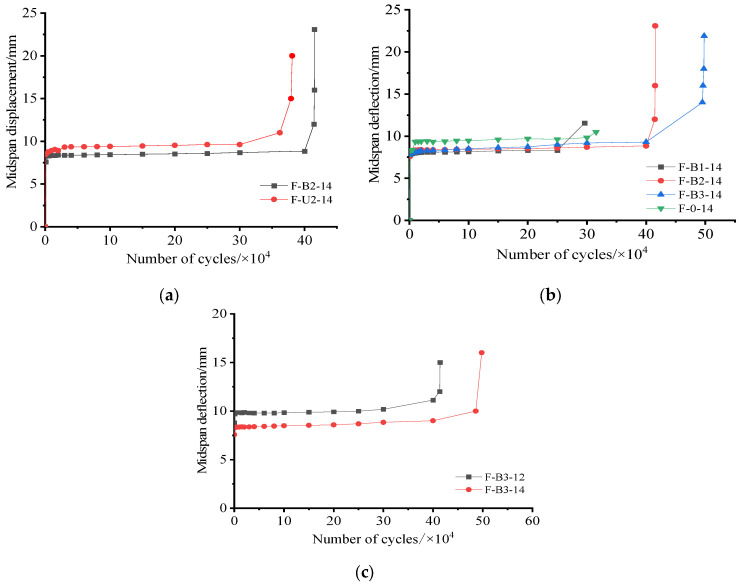
Number of cycles and mid-span deflection curves: (**a**) strengthening schemes; (**b**) textile ratio; (**c**) reinforcement ratio.

Figure 8a–c present the relationship of the midspan deflection development with the number of cycles for each tested beam. As shown in Figure 8, this relationship was characterized by three stages: rapid development (Stage I), stable development (Stage II), and destabilized development (Stage III). In Stage I, a large number of flexural cracks appeared, which led to a rapid reduction in TRC-strengthened beam stiffness. Therefore, deflection grew quickly in this stage. Stage I accounted for approximately 5–10% of the overall fatigue life. In Stage II, the number of cracks in the tested beam hardly increased, and cracks spread slowly, which resulted in the gradual degradation of the tested beam’s stiffness. Thus, beam deflection slightly grew as the number of cycles increased. This stage represented approximately 85% to 90% of the overall fatigue life. In Stage III, cracks rapidly spread, and stiffness swiftly degraded. This resulted in rapid deflection growth in this stage, which represented less than 5% of the overall fatigue life. In some of the tested beams, Stage III was so short that it could be neglected.

Figure 8b shows the variations in midspan deflection with the number of cycles for specimens with different textile ratios. As can be seen, Stage III was not clearly observed for F-0-14 and F-B1-14, which suggested that there was no obvious process whereby the deflection of non-strengthened and strengthened beams increased at low textile ratios. As shown in Figure 8b, the midspan deflections of TRC-strengthened beams were all slightly lower than those of non-strengthened beam F-0-14 for the same number of loading cycles. This proved that TRC reduced fatigue damage accumulation for RC beams. Figure 8c illustrates the curves of the midspan deflection versus the number of cycles for F-B3-12 and F-B3-14, both of which were prepared with three textile layers. As can be seen, Stage II of TRC-strengthened beams was extended by increasing the reinforcement ratio, which meant that the beam’s fatigue life was prolonged.

### 3.4. Residual Deflection

**Figure 9:**
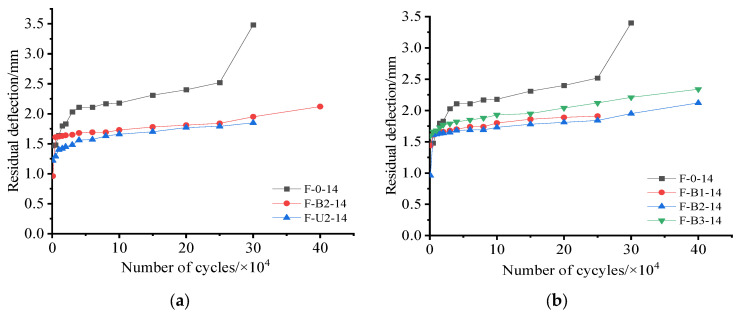

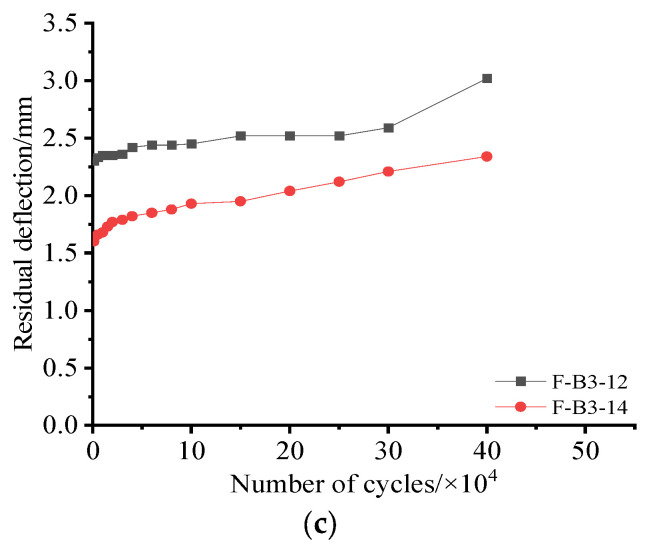
Residual deflection: (**a**) effect of strengthening methods; (**b**) effect of textile ratio; (**c**) effect of reinforcement ratio.

Figure 9a shows the residual deflection of the tested beams with different strengthening schemes (F-B2-14 and F-U2-14) versus the number of cycles. As can be seen, the residual deflection of F-B2-14 was slightly greater than that of F-U2-14 for the same number of cycles, which indicated that the U-wrapped strengthening method was marginally better than the single-side strengthening method in terms of reducing the cumulative growth of residual deflection. Figure 9b shows the residual deflection of tested beams with different textile ratios versus the number of cycles. As shown in Figure 9b, the residual deflection of F-B1-14 was larger than that of F-B2-14 for the same cycle number. However, it can also be seen that the residual deflection of F-B3-14 was greater than that of F-B1-14 under the same number of cycles. This phenomenon suggests that the residual deflection of the TRC-strengthened beam did not decrease further as the textile ratio increased. As this study used a limited number of samples, further research is required in this direction. However, Figure 9a,b show that the residual deflection of TRC-strengthened beams was significantly smaller than those of non-strengthened beams under the same number of cycles (Stage II), regardless of whether the beams were strengthened on one side or three sides, which also indicated that the application of TRC better decelerated the cumulative development of residual deflection in RC beams. Figure 9c shows the residual deflection of tested beams with different reinforcement ratios versus the number of cycles. As can be seen, the residual deflection of F-B3-12 was greater than that of F-B3-14 under the same cycle number, which indicated that increasing the reinforcement ratio better decelerated the cumulative residual deflection development in TRC-strengthened beams.

### 3.5. Load–Strain Response

For specimens F-0-14 and F-B1-14, steel bar and concrete strains were not recorded because the strain gauge did not work. Therefore, the data of these two test beams are not included in the following analysis.

**Figure 10:**
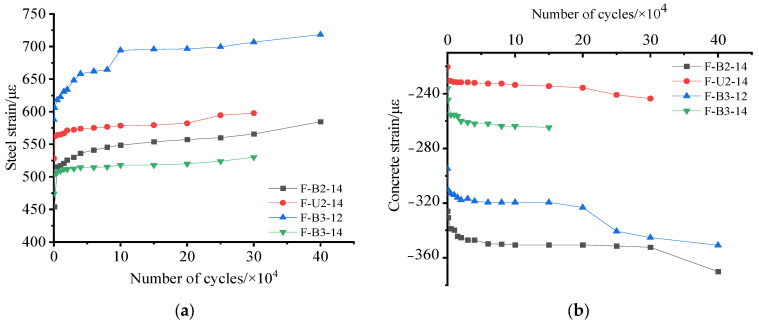
Load–strain response: (**a**) the relationship between steel strain and number of cycles; (**b**) the relationship between concrete strain and number of cycles.

Figure 10a shows the development of steel strain for each tested beam versus the number of cycles. As presented in Figure 10a, after the stable stage, the steel strain in F-U2-14 was greater than that in F-B2-14 for the same cycle number, which indicated that the single-side strengthening method better shared the stress load in steel and thus extended the fatigue life of the strengthened beams, compared with the U-wrapped strengthening method. This phenomenon was also attributed to the fact that the mechanical properties of the textile on the side of the U-wrapped beam were not fully utilized. In addition, as shown in Figure 10a, the steel strain in F-B3-14 was lower than that in F-B2-14 for the same cycle number, which indicated that steel strain was further decreased as the textile ratio increased. 

Figure 10b shows a plot of the concrete strain development versus the number of cycles for each tested beam. As can be seen, the absolute value of the concrete strain of specimen F-B3-14 was lower than that of F-B2-14 for the same cycle number, which indicated that the increase in textile ratio further reduced the concrete strain in the compression zone. Additionally, it was observed that the concrete strain in F-B3-14 was greater than that in F-B3-12 for the same loading cycle number, which indicated that the concrete strain in the compression zone was further reduced by increasing the textile ratio. The above analyses revealed that the accumulation of fatigue-related strain damage in beams could be decelerated by increasing both the textile and reinforcement ratios.

## 4. Conclusions

From this research, the following conclusions were drawn:

(1) The TRC-strengthened RC beams’ fatigue failure mode was affected by the textile ratio. When the textile ratio was less than 0.036%, the TRC-strengthened beam’s failure was characterized by textile snapping and concrete crushing but not steel fracture. When the textile ratio was greater than 0.036%, the TRC-strengthened beam’s failure was characterized by textile snapping and steel fracture but not concrete crushing.
(2) Both the U-wrapped and single-side strengthening methods improved the RC beam’s fatigue life. When the textile ratio is same, the single-side strengthening method performed better than the U-wrapped strengthening method in terms of improvement in the RC beam’s fatigue life. The TRC-strengthened RC beam’s fatigue life was increased with the textile and reinforcement ratios.
(3) The TRC-strengthened beam’s midspan deflection was characterized by three stages of development as the number of cycles increased: rapid development stage (Stage I), stable development stage (Stage II), and destabilized development stage (Stage III). Specifically, Stage II accounted for 85–90% of the overall fatigue life. The third stage of development was not obvious when the textile ratio of the TRC-strengthened beams was lower than 0.018%.
(4) The fatigue damage accumulation rate of TRC-strengthened beam can be decreased by increasing textile ratio or reinforcement ratio, which extend the fatigue life of RC beam.

## Figures and Tables

**Table 1:** The information of specimens.

Specimen	Strengthening Method	d/mm	ρ_s_	Layers of Textile	ρ_t_
M-0-12	Non-strengthened	12	0.95%	-	-
M-0-14	Non-strengthened	14	1.30%	-	-
F-0-14	Non-strengthened	14	1.70%	-	-
F-B1-14	Single-side	14	1.70%	1	0.018%
F-B2-14	Single-side	14	1.70%	2	0.036%
F-U2-14	U-wrapped	14	1.70%	3	0.036%
F-B3-12	Single-side	12	0.95%	3	0.054%
F-B3-14	Single-side	14	1.30%	3	0.054%

Notes: d means the diameter of steel bar; ρ_s_ means reinforcement ratio; ρ_t_ means textile ratio, ρ_t_ = A_0_/bh_0_, A_0_ is the total cross-sectional area of textile, b is the width of TRC-strengthened beam, h_0_ is the height from joint action point of steel bar to the top of TRC-strengthened beam.

**Table 2:** Mechanical properties of tensile steel.

Diameter of Rebar/mm	12	14
Average yield strength/MPa	546	500
Average tensile strength/MPa	639	635
Average elongation	21.67%	28.1%

**Table 3:** Mechanical properties of fiber yarns of textile.

Fiber Type	Number of Filaments per Bundle	Filament Tensile Strength (MPa)	Elastic Modulus (GPa)	Ultimate Strain	Quality of Unit Length (Tex)	Density (g/cm^3^)
Toray carbon	12k	4660	231	0.023	801	1.78
E-glass	4k	3200	65	0.045	600	2.58

**Table 4:** High-performance concrete mix (kg/m^3^).

Cement	Fly Ash	Silica Fume	Water	Fine Sand	Coarse Sand	Superplasticizer
475	168	35	262	460	920	9.1

**Table 5:** The test results of each specimen.

Num.	*F*_max_/kN	*F*_min_/kN	Failure Modes	*N*_u_/×10^4^
F-0-14	59.5	10.5	A	31.55
F-B1-14	59.5	10.5	B	29.62
F-B2-14	59.5	10.5	B	41.53
F-U2-14	59.5	10.5	B	38.10
F-B3-12	49.0	9.8	C	41.42
F-B3-14	59.5	10.5	C	49.80

Notes: *F*_max_ is the maximum fatigue load. *F*_min_ is the minimum fatigue load. *N*_u_ is fatigue life. “A” means the fatigue failure mode of concrete in the compression zone crushing and one tensile steel bar not rupturing. “B” means the fatigue failure mode of textile snapping and concrete in the compression zone crushing, but the tensile steel bar not rupturing. “C” means that textile snapped, and one tensile steel bar ruptured, but concrete in the compression zone did not crush.

## Data Availability

Not applicable.

## References

[1] Nie J., Wang Y., Cai C.S. (2011). Experimental Research on Fatigue Behavior of RC Beams Strengthened with Steel Plate-Concrete Composite Technique. J. Struct. Eng..

[2] Al-Saadi N., Mohammed A., Al-Mahaidi R., Sanjayan J. (2019). A state-of-the-art review: Near-surface mounted FRP composites for reinforced concrete structures. Constr. Build. Mater..

[3] Shaw I., Andrawes B. (2017). Repair of damaged end regions of PC beams using externally bonded FRP shear reinforcement. Constr. Build. Mater..

[4] Choobbor S.S., Hawileh R.A., Abu-Obeidah A., Abdalla J.A. (2019). Performance of Hybrid Carbon and Basalt FRP Sheets in Strengthening Concrete Beams in Flexure. Compos. Struct..

[5] Zhu Z., Zhu E., Ni Y., Li D. (2019). Flexural fatigue behavior of large-scale beams strengthened with side near surface mounted (SNSM) CFRP strips. Eng. Struct..

[6] Raoof S.M., Bournas D.A. (2017). TRM versus FRP in flexural strengthening of RC beams: Behaviour at high temperatures. Constr. Build. Mater..

[7] Scheerer S., Zobel R., Müller E., Senckpiel-Peters T., Schmidt A., Curbach M. (2019). Flexural Strengthening of RC Structures with TRC—Experimental Observations, Design Approach and Application. Appl. Sci..

[8] Yin S.P., Na M.W., Yu Y.L., Wu J. (2017). Research on the flexural performance of RC beams strengthened with TRC under the coupling action of load and marine environment. Constr. Build. Mater..

[9] Koutas L.N., Tetta Z., Bournas D.A., Triantafillou T.C. (2019). Strengthening of concrete structures with textile reinforced mortars: State-of-the-art review. J. Compos. Constr. ASCE.

[10] Zhu D., Liu S., Yao Y., Li G., Du Y., Shi C. (2019). Effects of short fiber and pre-tension on the tensile behavior of basalt textile reinforced concrete. Cem. Concr. Compos..

[11] Ebead U., Shrestha K.C., Afzal M.S., El Refai A., Nanni A. (2017). Effectiveness of fabric-reinforced cementitious matrix in strengthening reinforced concrete beams. J. Compos. Constr. ASCE.

[12] Larbi A.S., Contamine R., Ferrier E., Hamelin P. (2010). Shear strengthening of RC beams with textile reinforced concrete (TRC) plate. Constr. Build. Mater..

[13] Verbruggen S., Tysmans T., Wastiels J. (2016). Bending crack behaviour of plain concrete beams externally reinforced with TRC. Mater. Struct..

[14] Yin S., Xu S., Lv H. (2014). Flexural Behavior of Reinforced Concrete Beams with TRC Tension Zone Cover. J. Mater. Civ. Eng. ASCE.

[15] Calabrese A.S., Colombi P., D’Antino T. (2019). Analytical solution of the bond behavior of FRCM composites using a rigid-softening cohesive material law. Compos. Part B-Eng..

[16] Sneed L.H., Verre S., Carloni C., Ombres L. (2016). Flexural behaviour of RC beams strengthened with steel-FRCM composite. Eng. Struct..

[17] Raoof S.M., Koutas L.N., Bournas D.A. (2017). Textile-reinforced mortar (TRM) versus fibre-reinforced polymers (FRP) in flexural strengthening of RC beams. Constr. Build. Mater..

[18] Yin S., Jing L., Lv H. (2019). Experimental analysis of bond between corroded steel bar and concrete confined with textile reinforced concrete. J. Mater. Civ. Eng. ASCE.

[19] Sheng J., Wang L., Yin S. (2021). Study on the mechanical performance of TRC-strengthened RC beams under a salt freeze–thaw environment. Cold Reg. Sci. Technol..

[20] Pino V., Hadad H.A., Basalo F.D.Y., Nanni A., Ebead U.A., El Refai A. (2017). Performance of FRCM-strengthened RC beams subject to fatigue. J Bridge Eng. ASCE.

[21] Hadad H.A., Nanni A., Ebead U.A., El Refai A. (2018). Static and fatigue performance of FRCM-strengthened concrete beams. J. Compos. Constr. ASCE.

[22] Aljazaeri Z.R., Myers J.J. (2017). Fatigue and Flexural Behavior of Reinforced-Concrete Beams Strengthened with Fiber-Reinforced Cementitious Matrix. J. Compos. Constr. ASCE.

[23] Myroslava H. (2018). Peculiarities of bond strength degradation in reinforced concrete induced by accelerated electrochemical methods. Proced. Struct. Integr..

[24] Elghazy M., El Refai A., Ebead U., Nanni A. (2018). Fatigue and monotonic behaviors of corrosion- damaged reinforced concrete beams strengthened with FRCM composites. J. Compos. Constr. ASCE.

[25] Bressan J., Ghrib F., El Ragaby A. (2022). FRCM Strengthening of Corrosion-Damaged RC Beams Subjected to Monotonic and Cyclic Loading. J. Compos. Constr. ASCE.

[26] D’Antino T., Carloni C., Sneed L.H., Pellegrino C. (2015). Fatigue and post-fatigue behavior of PBO FRCM-concrete joints. Int. J. Fatigue.

[27] Peng H., Zhang J., Shang S., Liu Y., Cai C.S. (2016). Experimental study of flexural fatigue performance of reinforced concrete beams strengthened with prestressed CFRP plates. Eng. Struct..

